# Clinico-cytopathological subcategorization in thyroid nodules of atypia of undetermined significance/follicular lesion of undetermined significance using the TIRADS and Bethesda classifications

**DOI:** 10.3389/fendo.2023.1135196

**Published:** 2023-05-29

**Authors:** Amirhesam Babajani, Saeed Rahmani, Masoomeh Raoufi, Elham Shaarbaf Eidgahi, Amirreza Vahid Dastjerdi, Poya Behfarnia, Shayesteh Khalili, Noushin Afshar Moghaddam

**Affiliations:** ^1^ Oncopathology Research Center, Department of Molecular Medicine, School of Medicine, Iran University of Medical Sciences, Tehran, Iran; ^2^ Department of Pathology, School of Medicine, Imam Hossein Hospital, Shahid Beheshti University of Medical Sciences, Tehran, Iran; ^3^ Department of Radiology, School of Medicine, Imam Hossein Hospital, Shahid Beheshti University of Medical Sciences, Tehran, Iran; ^4^ Kidney Transplantation Complication Research Center, Mashhad University of Medical Sciences, Mashhad, Iran; ^5^ School of Medicine, Isfahan University of Medical Sciences, Isfahan, Iran; ^6^ Department of Internal Medicine, School of Medicine, Imam Hossein Hospital, Shahid Beheshti University of Medical Sciences, Tehran, Iran

**Keywords:** thyroid nodule, thyroid neoplasms, fine-needle aspiration, ultrasonography, cytology, Bethesda, AUS/FLUS, TIRADS

## Abstract

**Introduction:**

Bethesda category III – atypia of undetermined significance/follicular lesion of undetermined significance (AUS/FLUS) is a heterogeneous class of the Bethesda system for thyroid nodules. In order to clarify the therapeutic road for clinicians, this category was subclassified based on the cytopathological features. In this study, we evaluated the risk of malignancy, surgical outcome, demographic characteristics, and correlation of ultrasound features with the final outcome in patients with thyroid nodules based on AUS/FLUS subclassification.

**Method:**

After evaluating 867 thyroid nodules from three different centers, 70 (8.07%) were initially diagnosed as AUS/FLUS. The cytopathologists re-interpreted the FNA samples and subclassified them into five subcategories: architectural atypia, cytologic atypia, cytologic and architectural atypia, and Hürthle cell AUS/FLUS, and atypia, which was not specified. Based on the suspicious ultrasound features, an appropriate ACR TI-RADS score was allocated to each nodule. Finally, the malignancy rate, surgical outcomes, and ACR TI-RADS scores were evaluated among Bethesda category III nodules.

**Results:**

Among the 70 evaluated nodules, 28 (40%) were subclassified as Hürthle cell AUS/FLUS, 22 (31.42%) as cytologic and architectural atypia, 8 (11.42%) as architectural atypia, 7 (10%) as cytologic atypia, and 5 (7.14%) as atypia which was not specified. The overall malignancy rate was 34.28%, and the architectural atypia and Hürthle cell nodules displayed lower malignancy compared to other groups (P-Value<0.05). Utilizing ACR TI-RADS scores showed no statistical significance between Bethesda III subcategorization and ACR TI-RADS scores. However, ACR TI-RADS can be a reliable predictor for Hürthle cell AUS/FLU nodules.

**Conclusion:**

ACR TI-RADS helps evaluate malignancy only in the Hürthle cell AUS/FLUS subcategory of AUS/FLUS. Besides, cytopathological reporting based on the suggested AUS/FLUS subclassification could help clinicians take appropriate measures to manage thyroid nodules.

## 1 Introduction

Fine-needle aspiration (FNA) plays a prominent role in managing and work-up of thyroid nodules by approximating the malignancy risk and aiding rational clinical decisions for surgery or observation. In order to provide a standard and uniform reporting system for the cytological evaluation of thyroid nodule FNAs, *the Bethesda System for Reporting Thyroid Cytopathology* (TBSRTC) released a reporting algorithm with six diagnostic categories for FNA specimens (1, 2). Among these six diagnostic categories, Bethesda category III – atypia of undetermined significance/follicular lesion of undetermined significance (AUS/FLUS) contains a heterogeneous population of thyroid lesions with a confusingly broad range of malignancy-risk ranging from 10%–30% impeding appropriate consideration for clinical management of patients (3). Therefore, evaluating the malignancy risk for an AUS nodule is problematic since only a subset of patients with AUS nodules have a surgical follow-up. The patients undergoing thyroid resection are a selected population with repeated AUS results or complex clinical or imaging findings. Based on the TBSRTC, 20–25% of this population has cancer after surgery; however, this is undoubtedly overestimated (4, 5). The viable clinical approaches to AUS/FLUS thyroid nodules consist of molecular testing (if available), repeated FNA, and diagnostic surgery with attention to thyroid-stimulating hormone (TSH) levels (6).

Based on the 2nd ed. TBSRTC, AUS/FLUS species are mainly described as thyroid lesions with a comparable proportion of macro-and micro follicles, mild nuclear atypia, wide-ranging oncocytic alteration, and poor fixation (7). Although a prominent population of micro follicles in the FNA sample exists, it does not fulfil the criteria for follicular neoplasm/suspicious for follicular neoplasm. The reason arises from the predominance of micro follicles in a sparse cellular aspirate with slight colloids (4, 5). Notably, AUS and FLUS are considered synonyms; thus, they should not be utilized to represent two different interpretations. In this regard, the 2017 TBSRTC suggests subcategorization for AUS/FLUS to avoid vague descriptors and provide appropriate risk clarification consisting of (a) cytologic atypia, (b) architectural atypia, (c) cytologic and architectural atypia, (d) Hürthle cell AUS/FLUS, (d) atypia, not otherwise specified (1). It has been demonstrated that different atypia patterns of AUS/FLUS are associated with additional malignancy risk. For instance, nuclear atypia has been shown to represent a more prominent malignant risk compared to architectural atypia (8, 9). Therefore, considering other diagnostic approaches would improve risk assessment and patient care.

Several studies have made the possible role of thyroid ultrasonography (US) prominent in the management of AUS/FLUS thyroid nodules (10–12). Introducing Thyroid Imaging Reporting and Data System (TIRADS) by the American College of Radiologists (ACR) has provided a chance to compare the sonographic properties of thyroid nodules with cytological findings to discriminate among benign and malignant thyroid nodules (13). However, to the best of our knowledge, no study has described the correlation of US features (especially by considering TIRADS classification) of thyroid nodules to the Bethesda III subclassifications.

Herein, we have assessed the clinical outcomes of thyroid nodules which are diagnosed with Bethesda III considering its subclassification. Besides, the study has compared the properties of thyroid lesions in different Bethesda III subcategories regarding TIRADS imaging features in order to determine whether TIRADS features and AUS/FLUS subclassification can be used in patient management.

## 2 Materials and methods

### 2.1 Patients

The study was performed after approval by the Shahid Beheshti University of Medical Sciences Ethics Committee (Ethics Code: IR.SBMU.MSP.REC.1399.440) and after obtaining informed consent from the patients. This multicentered study was performed mainly in Imam Hossein Educational Hospital, Tehran, Iran, and the data includes FNAs performed at this medical center or outside FNAs interpreted by Imam Hossein Educational Hospital cytopathologists. Two reviewers assessed the records of all patients who underwent thyroid nodules FNA between September 2020 to January 2022 independently. In this regard, FNAs with AUS/FLUS reports were included. Besides, Bethesda III thyroid lesions with previous FNAs representing highly malignant risk (Bethesda Class IV-VI), as well as patients with incomplete documented follow-ups, were excluded. Among 867 screened nodules, 70 reports met the inclusion and exclusion criteria of the study. The demographic properties (age and sex), previous US reports, management plans, and outcomes were collected from the hospital information system (HIS) of evaluated centers. The minimum time for follow-up was six months.

### 2.2 Fine needle aspiration procedures

Radiologists of evaluated educational centers with at least five years of experience in FNA carried out the procedures. Based on the patient’s priority, FNAs were performed with or without an anesthetic (e.g., lidocaine) and repetitive movements of a 25-gauge needle attached to a 2 mL syringe within the nodules under US guidance. The aspirates within the syringe were smeared on appropriate glass slides and immediately fixed with 95% alcohol for Papanicolaou and/or Wright-Giemsa staining.

### 2.3 Cytopathologic analysis

**Figure 1:**
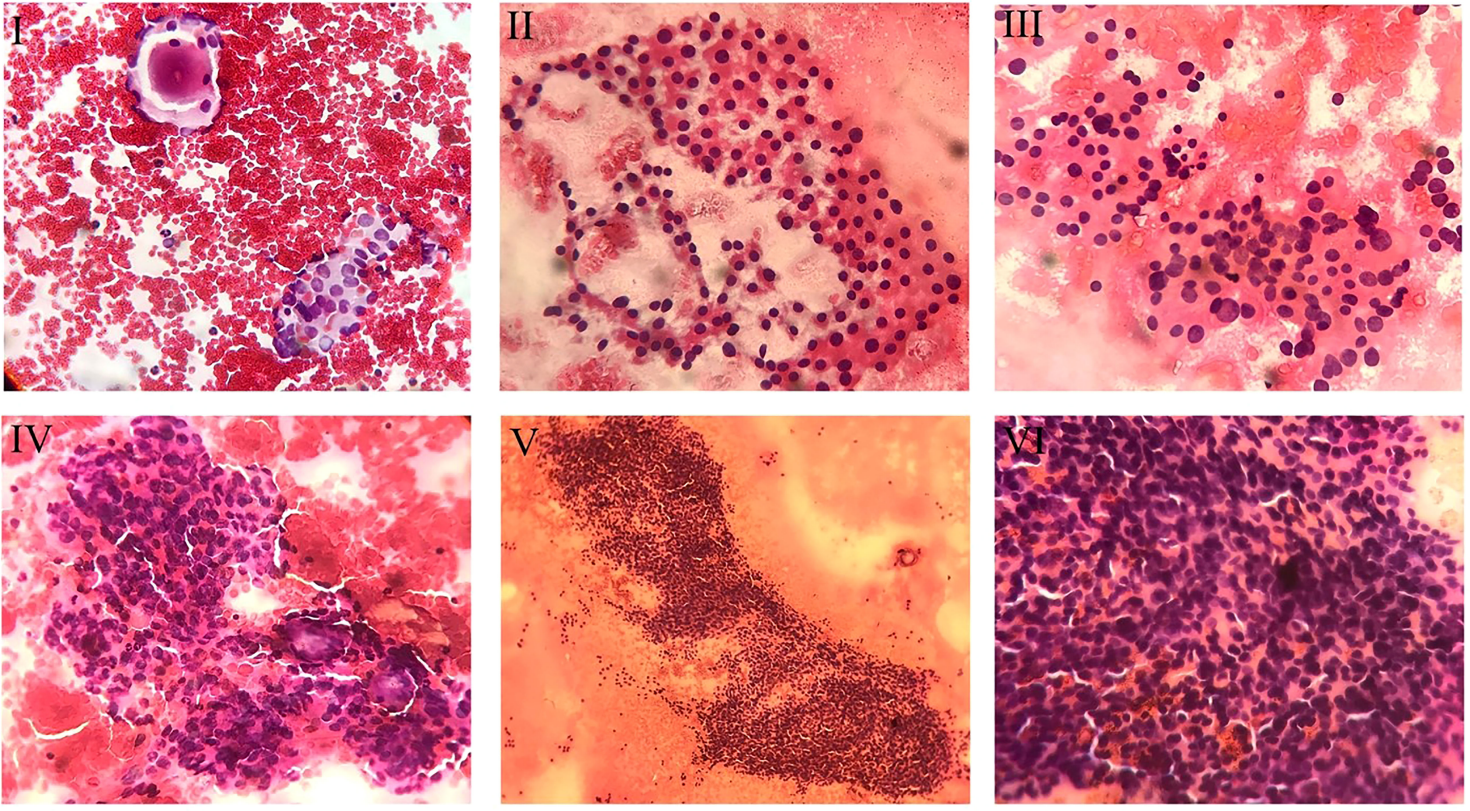
Cytopathologic view of different subcategories of AUS/FLUS specimens. I. Cytologic and Architectural Atypia. In the upper left corner, one micro follicle, and in the lower right corner, cellular clusters with pale and enlarged nuclei mimicking papillary carcinoma are noted (Papanicolaou staining, x200). II. Hürthle Cell. Smear exclusively shows the Hürthle cells population (Papanicolaou staining, x200). III. Hürthle Cell and Cytologic atypia. The smear reveals oncocytic follicular cells (Hürthle cells) with some degree of nuclear enlargement (Papanicolaou staining, x200). IV. Architectueal Atypia. showing nuclear crowding and micro follicles (Papanicolaou staining, x200). V. Architectural Atypia. Cellular cluster with nuclear crowding and overlapping is noted (Papanicolaou staining, x100). VI. Architectural Atypia. The smear reveals clusters with nuclear crowding and overlapping (Papanicolaou staining, x400).

Four cytopathologists evaluated the interpretation of FNA samples from different centers independently. Besides, another professional cytopathologist assessed the interpreted slides and reviewed the challenging reports. The specimens were allocated into six categories regarding TBSRTC: (a) Bethesda I for nondiagnostic or unsatisfactory slides, (b) Bethesda II for benign lesions, (c) Bethesda III for AUS/FLUS, (d) Bethesda IV for follicular neoplasm or suspicious lesions, (e) Bethesda V for suspicious malignant lesions, and (f) Bethesda VI for malignant nodules (14). After collecting AUS/FLUS specimens, these slides were evaluated and subclassified into five categories based on cytopathological features (**Figure 1**): (a) Architectural atypia for sparse cellularity along with crowded follicular cells present in trabecular and/or microfollicular positionings, (b) Cytologic atypia for focal, extensive, mild nuclear alteration and/or atypical cyst lining cells, (c) Cytologic and architectural atypia representing with both cytologic atypia and architectural atypia which are discordant, (d) Hürthle cell AUS/FLUS for spare cellular aspirates with exclusive Hürthle cells, (e) Atypia which was not specified (5).

### 2.4 Thyroid ultrasound examination and interpretation

**Figure 2:**
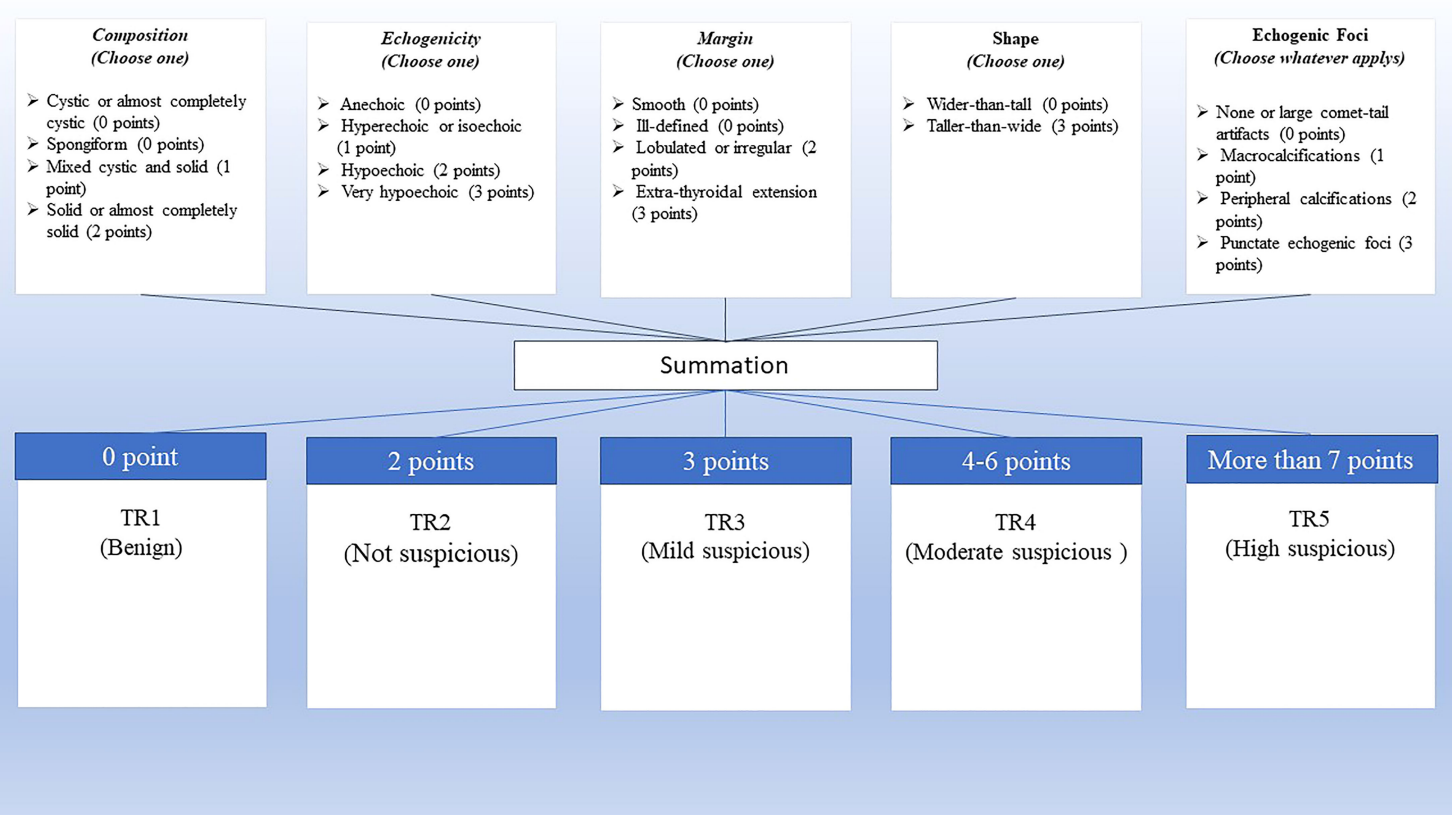
ACR TI-RADS scoring. Scoring is calculated based on the five categories of US findings (upper row). A higher cumulative score shows a higher TI-RADS level and a higher probability of malignancy (lower row).

The trained radiologists independently performed the US examinations in evaluated educational centers with at least five years of experience. Besides, another professional radiologist assessed and re-scored the challenging reports. Radiographic features of thyroid nodules were reported regarding ACR TI-RADS comprising: (a) composition, which describes the internal components of a nodule, (b) echogenicity, which shows echogenicity of the nodule components relative to nearby thyroid tissue, (c) shape which describes length to width ratio in anteroposterior to horizontal diameter in the transverse plane, (d) margin which describes border of thyroid nodule and the adjacent thyroid parenchyma or extrathyroidal structures, and (e) echogenic foci defined as markedly enhanced echogenicity of focal regions within a nodule compared to the nearby tissues. The nodules were classified into five TIRADS, including benign (TR1), not suspicious (TR2), mildly suspicious (TR3), moderately suspicious (TR4), and highly suspicious (TR5) (**Figure 2**) (15).

### 2.5 Statistical analysis

Our standard data reference consists of FNA reports and permanent section results of surgeries. Nodules with surgical statements declaring neoplasm and/or radioactive iodine (RAI) were considered malignant. In order to describe normal variables, the mean ± standard deviation and for abnormal variables, the Median (IQR) were used. Besides, to compare the variables between the two groups, in the case of normal variables, the independent t-test and, for others, the Mann-Whitney test were used. The Kruskal-Wallis test was used to comparing between more than two groups. The Chi-square test or Fisher’s exact test was used in order to compare the categorical variables. P values lower than 0.05 were measured as statistical significance.

## 3 Results

The mean age of the 70 patients was 46.71 ± 13.52 years ranging from 21 to 83 years. Of the 70 patients who met the appropriate criteria to be included in the study, 60 (85.7%) were women, and 10 (14.3%) were men. In this regard, there is no significant age difference between men and women (P-value = 0.42).

Of the 867 screened thyroid nodules, 76 (8.76%) were initially diagnosed as AUS/FLUS based on the TBSRTC. However, six were excluded due to previous FNAs representing highly malignant risk or incomplete documented follow-up. The AUS/FLUS thyroid lesions were classified based on the Bethesda subcategorization described in the method section. Of 70 AUS/FLUS thyroid nodules, 7 (10%) were subcategorized as cytologic atypia, 8 (11.42%) as architectural atypia, 22 (31.42%) as cytologic and architectural atypia, 28 (40%) as Hürthle cell AUS/FLUS, and 5 (7.14%) as atypia which was not specified. Evaluating the thyroid nodule size showed a statistical significance between nodule size and Bethesda III subcategorization (P-Value<0.05). In this regard, the mean size of thyroid nodules was significantly larger in architectural atypia compared to cytologic atypia (P-Value<0.05) (**Table 1**).

**Table 1:** Nodule size among subcategories of Bethesda III.

Bethesda Subsection	Nodule size (mm)	[min,max]
Architectural	34.42 ± 15.32	[12.40,55.00]
Cytologic	16.68 ± 16.36	[7.00,53.00]
Cytologic and architectural	23.90 ± 16.29	[5.00,73.00]
Hürthle cell AUS/FLUS	18.63 ± 9.99	[3.00,43.00|
Not specified	16.56 ± 13.93	[8.00,41.00]

However, the comparison among other groups did not reveal any significant differences. Although the results demonstrated that larger nodules are more suspicious to be malignant overall (P-Value<0.05), comparing the nodule size among different subcategories indicated that there is a statistical significance between nodule size and malignancy only in cytologic and architectural atypia subcategory (P-Value<0.05). **Table 2** summarizes the demographic differences, including age, sex, and size, between malignant and benign nodules among five subcategories of AUS/FLUS. The results showed no significant difference in age, sex, and risk of malignancy.

**Table 2:** Demographics of thyroid nodules based on the AUS/FLUS subcategories.

	Architectural			Cytologic			Cytologic and architectural			Hürthle cell AUS/FLUS			Not specified		
	Benign	Malignant	P	Benign	Malignant	P	Benign	Malignant	P	Benign	Malignant	P	Benign	Malignant	P
Age*															
	43.75 ± 14.98	–	–	42.67 ± 8.96	40.75 ± 10.53	0.81	51.11 ± 13.74	41.46 ± 12.99	0.11	49.95 ± 13.42	44.50 ± 6.25	0.35	50.00 ± 28.79	57.00 ± 8.48	0.77
Size*															
	34.42 ± 15.32	–	–	11.33 ± 4.51	11.33 (7,15.8)	0.86	15.56 ± 8.34	29.68 ± 18.15	0.02	17.39 ± 10.36	23.17 ± 7.57	0.09	8.93 ± 0.90	28.00 ± 18.38	0.20
Gender*															
Men	4 (50%)	0	–	1 (33.30%)	0	0.43	1 (11.10%)	0	0.41	2 (9.10%)	0	0.60	1 (33.30%)	1 (50%)	0.70
Women	4 (50%)	0		2 (66.70%)	4 (100%)		8 (88.90%)	13 (100%)		20 (90.90%)	6 (100%)		2 (66.70%)	1 (50%)	

*Normal data are reported with mean ± standard deviation and non-normal data with median (IQR).

The flow-up documentation showed that among 70 patients, 41 (58.57%) underwent surgery as the final therapeutic decision, while 29 (41.42%) performed repeated follow-up FNAs (active surveillance), which did not convince physicians to use surgery as the therapeutic choice. Among the patient with total or partial thyroid lobectomy, the permanent section analysis showed 16 (39.02%) benign and 25 (60.97%) malignant thyroid nodules. Malignancy rates among these five subcategories of AUS/FLUS showed that there is a statistical significance between the malignancy rate and Bethesda III subcategorization (P-Value <0.05). In this regard, the nodules subclassified into Hürthle cell AUS/FLUS and architectural atypia possess lower malignancy risk compared to other subclassifications (P-Value<0.05) (**Table 3**).

**Table 3:** Malignancy risk in different Bethesda III subcategories.

Bethesda III subcategory	Benign	Malignant	Chi-Square Test
Architectural	8/8 (100%)	0/8 (0%)	Value= 4.50 P-Value= 0.03
Cytologic	3/7 (42.85)	4/7 (57.14%)	Value= 0.14 P-Value= 0.70
Cytologic and architectural	9/22 (40.90%)	13/22 (59.09%)	Value= 0.73 P-Value= 0.39
Hürthle cell AUS/FLUS	22/28 (78.57%)	6/28 (21.42%)	Value= 9.14 P-Value= 0.002
Not specified	3/5 (60.00%)	2/5 (40%)	Value= 0.20 P-Value= 0.65

**Figure 3:**
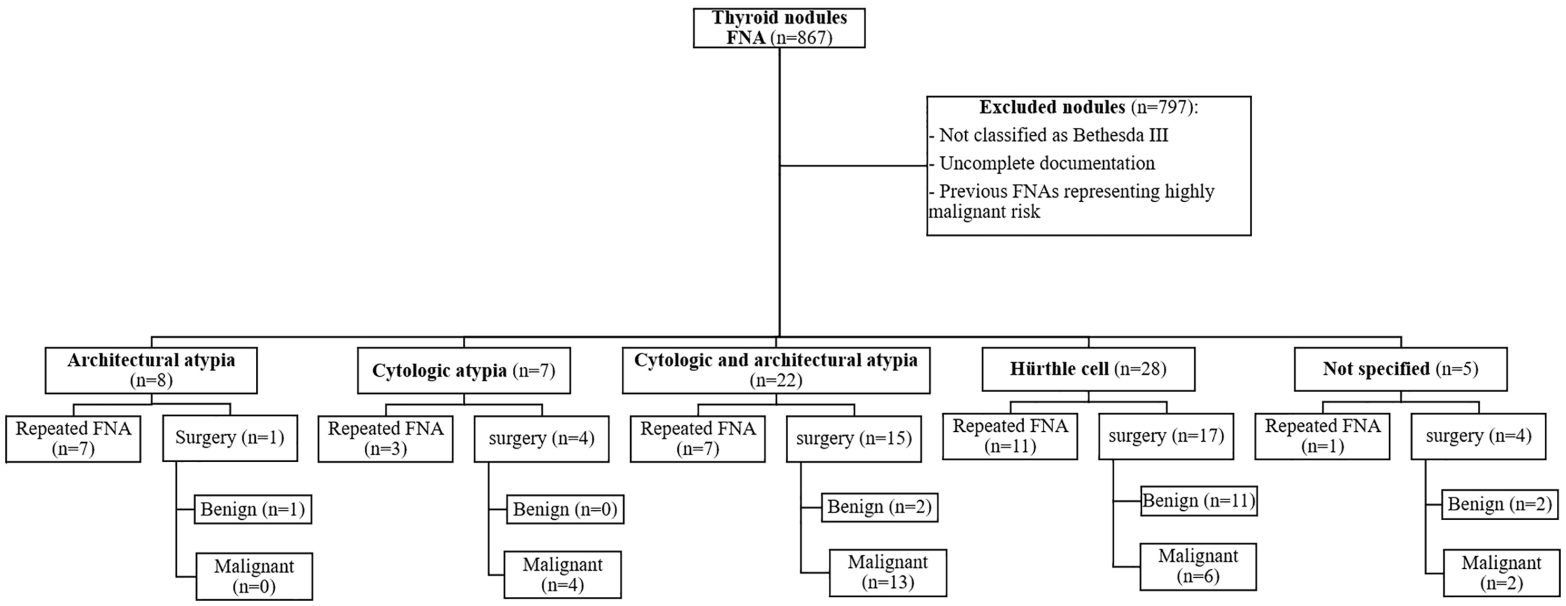
Diagram of clinical outcome of thyroid nodules. Among 867 evaluated thyroid nodules, 70 lesions were sub-categorized into architectural atypia, cytologic atypia, cytologic and architectural atypia, Hürthle cell AUS/FLUS, and atypia, not otherwise specified. The outcomes are shown based on surgery or follow-up US-FNA.

Evaluating the pathology reports of resected thyroid among patients who underwent surgery revealed that the final diagnoses consisted of papillary thyroid carcinoma (PTC) (51.21%), lymphocytic thyroiditis+multinodular goiter (12.19%), lymphocytic thyroiditis (9.75%), multinodular goiter (MNG) (9.75%), Hürthle cell adenoma (4.87%), Hürthle cell carcinoma (4.87%), and non-invasive follicular thyroid neoplasm with papillary like nuclear features (NIFTP) (2.43%). **Figure 3** shows the subcategorization of AUS/FLUS thyroid lesions, clinical decisions, and final pathological outcomes.

In order to compare US characteristics between benign and malignant nodules described as ACR TI-RADS, the scores were evaluated in each subcategorization. The overall malignancy rates among ACR TI-RADS three, four, and five were 19.04%, 38.09%, and 71.42%, respectively. The analysis showed a statistical significance between ACR TI-RADS and malignancy rates (P-Value<0.05). However, no statistical significance was observed between Bethesda III subcategorization and ACR TI-RADS scores (P-Value>0.05). Evaluation of ACR TI-RADS scores among Bethesda III subcategorization regarding the malignancy rate showed that ACR TI-RADS scores statistically are predictive for malignancy only in Hürthle cell AUS/FLU subclassification (P-Value <0.05). Besides, ACR TI-RADS scores three and four were statistically different between malignant and benign nodules in architectural atypia subclassification (P-Value <0.05) (**Table 4**).

**Table 4:** Bethesda III subcategorization and ACR TI-RADS scores.

	Architectural			Cytologic			Cytologic and architectural			Hürthle cell AUS/FLUS			Not specified		
	Benign	Malignant	P	Benign	Malignant	P	Benign	Malignant	P	Benign	Malignant	P	Benign	Malignant	P
TR3	5	0	–	0	0	1.00	3	3	1.00	8	1	0.02	1	0	–
TR4	3	0	–	3	3	1.00	5	9	0.28	13	2	0.005	2	2	1.00
TR5	0	0	1.00	0	1	–	1	1	1.00	0	4	–	0	0	1.00
Fisher’s Exact Test	P_Value =0.72			P_Value=0.65			P_Value = 0.82			P_Value = 0.003			P_Value = 0.66		

## 4 Discussion

Several studies have reported an overall rate of malignancy ranging from 10% to 30% in the Bethesda III category of thyroid nodules (1, 3, 16, 17). Besides, TBSRTC 2nd ed revealed a higher risk of malignancy AUS/FLUS thyroid nodules (10-30%) compared to 1^st^ edition (5-15%), showing the heterogeneous nature and outcomes of these nodules. The 2015 American Thyroid Association (ATA) recommend active surveillance, including repeated FNA, molecular testing (like ThyroSeq v.3), or diagnostic lobectomy after considering worrying clinical and sonographic features along with patient preference and feasibility (6). This fact has resulted in severe challenges in patient management and treatment planning. In order to increase the transparency and efficacy of AUS/FLUS thyroid nodules, TBSRTC has introduced a subclassification for the Bethesda III category, which consists of five subcategories based on the cytopathologic interpretation (1, 5). To evaluate clinical approaches, risk of malignancy, and outcome in AUS/FLUS thyroid nodules, we assessed the thyroid nodules regarding the suggested subcategorization of AUS/FLUS. Our study showed that Hürthle cell AUS/FLUS is the most classified lesion, while cytologic and architectural atypia, architectural atypia, cytologic atypia, and atypia were not specified are in the second to fifth positions, respectively. However, considering the malignancy risk, Hürthle cell AUS/FLUS and architectural atypia showed lower malignancy risk compared to other subcategorizations. There are controversial reports on the prevalence of each subclassification and its risk of malignancy. Guleria et al. have shown that cytologic and architectural atypia was the most classified AUS/FLUS lesions, followed by Hürthle cell AUS/FLUS, architectural atypia, atypia which was not specified, and cytologic atypia. Also, they showed that cytologic atypia lesions showed a higher risk of malignancy (18). A meta-analysis by Valderrabano et al. also demonstrated that the malignancy rates were lower for architectural atypia and oncocytic atypia (Hürthle cell). Evaluating the final pathology reports of patients who underwent surgery showed that PTC is the most common diagnosis among AUS/FLUS nodules. In accordance with our study, it has been reported that PTC is the most frequently diagnosed malignancy for AUS/FLUS nodules (19). It seems that nodule size alteration, US follow-up findings, and biochemistry profile persuade clinicians to take surgery as a final therapeutic approach. Reviewing the final diagnosis among the Bethesda III subcategories showed that the Hürthle cell AUS/FLUS as a subcategory and NIFTP as a rare diagnosis are challenging for clinicians and cytopathologists.

As a challenging subcategory of AUS/FLUS, the Hürthle cells are characterized as oncocytes associated with the thyroid epithelial cells displaying plentiful fine cytoplasmic granules around the nucleus due to the presence of oversize, vacuolated, and dilated mitochondria (20, 21). It has been shown that Hürthle cells can be found both in non-malignant lesions such as Hashimoto’s disease, nodular goiter, Graves’ disease, radiotherapy or chemotherapy-associated lesions, and also thyroid neoplasms including Hürthle cell adenomas, Hürthle cell carcinomas, follicular thyroid carcinomas (FTC), follicular thyroid adenomas (FTA), and PTC (22). The presence of Hürthle cells in thyroid FNAs persuades cytopathologists to discriminate nonneoplastic (mainly hyperplastic) from neoplastic lesions, that extensively clarifies clinical management approaches.

Based on the BSRTC, the FNA reports of Hürthle cells are primarily classified into the category III or IV (1). An exclusively Hürthle cell specimen can be categorized as AUS/FLUS in patients with MNG and lymphocytic (Hashimoto) thyroiditis, generally considered hyperplastic rather than neoplastic (23). Notably, most malignancies in known Hashimoto thyroiditis patients are considered PTC (24). Thus, reporting Hürthle cell AUS/FLUS as the most common subcategory with various differential diagnoses should be considered carefully to provide detailed information to avoid a needless lobectomy.

Another critical challenge in AUS/FLUS subcategorization is NIFTP, a shallow-risk thyroid lesion mainly subclassified into architectural atypia or cytologic and architectural atypia. It has been shown that up to 20% to 25% of all lesions previously diagnosed as thyroid malignancies should have been categorized as NIFTP (25–27). Any of the six TBSRTC categories may precede a report of NIFTP; however, the most frequent NIFTP report is encountered in the setting of AUS/FLUS (28). NIFTPs have cytologic features similar to PTC except for a follicular architecture and classical papillae of PTC. Therefore, the presence of true papillae with fibrovascular cores and/or psammomatous calcifications will exclude NIFTP diagnosis. Considering the lower risk of malignancy in NIFTP, suspicious US pattern, indeterminate cytology, and *RAS* mutation in ThyroSeq should guide clinicians to NIFTP for considering less aggressive therapeutical approaches (18, 29). Besides, future studies are required to determine whether NIFTP is associated with specific patterns of AUS/FLUS. This may hypothetically persuade efforts to recognize NIFTPs in AUS/FLUS subcategories.

Nodular size is another prominent feature of thyroid nodules. There is a considerable discrepancy in the correlation between thyroid nodule size and malignancy risk. In this study, we have evaluated the size of thyroid nodules among different AUS/FLUS subcategorization. In this regard, nodules classified into architectural atypia (34.42 mm) were significantly larger than cytologic atypia (16.68 mm). Notably, our results showed that nodules classified into architectural atypia display lower malignancy risk than other categories, including those with cytologic atypia. These results are in accordance with previous studies that demonstrated thyroid nodules smaller than 20 mm have a higher malignancy risk than larger lesions (30, 31). On the other hand, comparing the size of malignant and benign nodules in each subclassification of AUS/FLUS showed that malignant nodules (29.68 mm) are larger than benign nodules (15.56 mm) only in the cytologic and architectural atypia subcategory. At the same time, the difference was not significant in other subclassifications. Some reports show that an increase in thyroid nodule size influences cancer risk in a nonlinear fashion with a threshold of 20 mm (32). In order to take appropriate clinical measures for AUS/FLUS thyroid nodules, Sengul et al. have recommended active surveillance for managing these thyroid nodules with a size of 10-15 mm (33). It seems that the size of thyroid nodules is not an accurate predictive feature for the malignancy risk of the thyroid nodules, and FNA cytology is required besides the size feature (34).

As another risk-evaluating tool, US characteristics have shown an acceptable predictive role in determining the malignancy of thyroid nodules. In this regard, some studies have assessed the predictive role of US features in AUS/FLUS nodules (35, 36). In order to simplify and standardize the evaluation and reporting of US characteristics of a thyroid nodule, the *American College of Radiology* has introduced the ACR TI-RADS reporting system consisting of five grades of malignancy susception (15). ACR TI-RADS has been utilized in evaluating thyroid nodules of different cytopathologic categories and has proven its efficacy in clinical practice (37). However, the predictive role of ACR TI-RADS in AUS/FLUS subclassifications is unclear. In this study, we have shown that ACR TI-RADS is not predictive for Bethesda III subcategorization. Some studies have evaluated the TIRADS score between AUS and FLUS, which has revealed no significant differences between benign and malignant FLUS nodules, while there were significant differences between benign and malignant nodules of the AUS subcategory (2). On the other hand, we have shown that ACR TI-RADS scoring is significantly different between benign and malignant nodules only in Hürthle cell AUS/FLUS subclassification. Słowińska-Klencka et al. assessed the diagnostic effectiveness of EU-TIRADS for Hürthle cell thyroid nodules in Bethesda III-V. They concluded that EU-TIRADS would not assist clinicians in taking the appropriate measure in patients with thyroid Hürthle cell nodules, especially in the Bethesda IV classification. However, we have demonstrated that ACR TI-RADS can be used in Hürthle cell AUS/FLUS thyroid nodules to discriminate between benign and malignant nodules (38).

Our study has some limitations: 1) Although the ratio of AUS/FLUS reports among evaluated nodules was sensible according to previous reports, the number of AUS/FLUS reports in evaluated centers was low. 2) the unavailability of molecular pattering in evaluated centers may influence the final clinical decision in patients with AUS-FLUS nodules. 3) We tried recruiting several expert radiologists and cytopathologists; however, reporting US features and FNA slides is still subjective, and results vary interpretations among radiologists and cytopathologists.

## 5 Conclusion

In conclusion, we have demonstrated that Hürthle cell AUS/FLUS and architectural atypia showed lower malignancy among different AUS/FLUS subcategorization. Besides, Although ACR TI-RADS cannot be used in sub-categorizing AUS/FLUS lesions, it is predictive of malignancy in Hürthle cell AUS/FLUS subclassification.

## Data availability statement

The original contributions presented in the study are included in the article, further inquiries can be directed to the corresponding author (afsharmoghadam@sbmu.ac.ir).

## Ethics statement

The studies involving human participants were reviewed and approved by Shahid Beheshti University of Medical Sciences Ethics Committee (Ethics Code: IR.SBMU.MSP.REC.1399.440). The patients/participants provided their written informed consent to participate in this study.

## Author contributions

AB, SR, and NM conceived and designed the analysis AB, SR, AD, and NM collected the data MR, SK, PB, and NM contributed data or analysis tools AB and EE performed the analysis AB, and NM wrote the paper. All authors contributed to the article and approved the submitted version.
